# Production of Mouse Ultrasonic Vocalizations and Distress Calls Is Associated with Different Patterns of Fos Expression in the Nucleus Retroambiguus

**DOI:** 10.1523/ENEURO.0100-26.2026

**Published:** 2026-07-31

**Authors:** Patryk Ziobro, Da-Jiang Zheng, Aditya Rawal, Zizhan Zhou, Asmita Mittal, Katherine A. Tschida

**Affiliations:** Department of Psychology, Cornell University, Ithaca, New York 14853

**Keywords:** Fos, mouse, nucleus retroambiguus, premotor, squeak, ultrasonic vocalization

## Abstract

Animals produce different vocalization types, which differ in their acoustic features and are produced in different behavioral contexts. How vocalization-related brain circuits are organized to enable the production of different vocalization types remains poorly understood. The nucleus retroambiguus (RAm) is a hindbrain premotor region that regulates the production of both ultrasonic vocalizations (USVs) and distress calls (squeaks) in adult mice, but whether distinct or overlapping populations of RAm neurons are recruited during the production of these two vocalization types is unknown. In the current study, we used Fos immunohistochemistry to compare the counts and spatial distributions of Fos-positive RAm neurons in males and females that produced USVs and females that produced courtship squeaks. We also combined in vivo activity-dependent (TRAP2) labeling with Fos immunohistochemistry to directly compare Fos expression associated with the production of USVs and courtship squeaks in the same females. Our findings suggest that RAm contains three vocalization-related populations of neurons: squeak-related neurons, USV-related neurons, and shared neurons that are recruited during both vocalization types. These findings refine current models of the premotor control of vocalization and set the stage for future work to explore anatomical and functional heterogeneity within RAm.

## Significance Statement

Animals produce multiple vocalization types that differ in their acoustic features, but how the brainstem is organized to enable the production of different vocalization types remains unknown. In the current study, we performed immediate early gene mapping in the nucleus retroambiguus, a vocalization-related hindbrain region, to compare patterns of neural activity in mice that produced social calls versus distress calls. Our findings suggest that even in premotor regions, largely nonoverlapping sets of neurons may be recruited during the production of different vocalization types.

## Introduction

To communicate successfully, animals produce different vocalization types, which differ in their acoustic features and are produced in different behavioral contexts (e.g., contact calls, alarm calls, etc.). In mammals, vocalization requires an increase in expiratory airway pressure coordinated with the adduction of laryngeal muscles, and the resulting sound can be further influenced by vocal tract filtering. Thus, despite their differences in acoustic features, the production of different vocalization types involves the recruitment of laryngeal, respiratory, and orofacial muscles. How vocalization-related brain circuits are organized to enable the production of different vocalization types remains poorly understood.

The nucleus retroambiguus (RAm) is a hindbrain region that plays a well-established role in vocal production. RAm sends axonal projections to the nucleus ambiguus, which houses laryngeal motor neurons, as well as to the thoracic and lumbar spinal cord, which houses expiratory motor neurons (Miller et al., 1985; Merrill and Lipski, 1987; Holstege, 1989; Katada et al., 1996). RAm also receives a robust descending projection from the periaqueductal gray (PAG), a midbrain region that gates the production of innate vocalizations in mammals (Jürgens, 1994, 2002, 2009). Artificial activation of RAm-projecting PAG neurons elicits the production of vocalizations in mice (Tschida et al., 2019; Park et al., 2024), and work in decerebrate cats found that medulla transections at the level of RAm, as well as excitotoxic RAm lesions, block PAG-elicited vocalizations (Zhang et al., 1995; Shiba et al., 1997). Conversely, direct pharmacological stimulation of RAm elicits the production of vocalizations (Zhang et al., 1992, 1995; Subramanian and Holstege, 2009). These past studies support the important role of RAm in regulating vocal production but leave open the question of how RAm participates in the production of different vocalization types.

Two recent studies in mice have begun to shed light on this question. Mice produce two major vocalization types: (1) ultrasonic vocalizations (USVs), which are produced by adults during social interactions (Nyby, 1983; Holy and Guo, 2005; Moles et al., 2007; Warren et al., 2020; Zhao et al., 2021, 2025), and (2) distress calls (squeaks), which are produced in aversive behavioral contexts (Grimsley et al., 2013, 2016; Finton et al., 2017; Ruat et al., 2022; Ziobro et al., 2024). The first study used an activity-dependent strategy to express tetanus toxin light chain in RAm neurons that upregulated expression of the immediate early gene Fos following the production of courtship USVs in male mice (Park et al., 2024). Synaptic silencing of these Fos-defined RAm neurons blocked the production of both male courtship USVs and squeaks elicited by tail pinch, suggesting that at least some RAm neurons regulate both vocalization types. However, optogenetic activation of RAm-voc neurons elicited only USV production, suggesting that the activity of additional neurons is needed to drive the production of squeaks. A second study found that caspase-mediated ablation of neurotensin (*Nts*)-expressing RAm neurons blocked the production of male courtship USVs (Veerakumar et al., 2023). Notably, low-frequency optogenetic activation of RAm *Nts* neurons elicited the production of squeak-like (<20 kHz) vocalizations, while high-frequency optogenetic activation elicited the production of vocalizations in the ultrasonic range. Together, these studies show that RAm regulates the production of both USVs and squeaks, but the degree to which overlapping versus distinct sets of RAm neurons are recruited during the two vocalization types remains unclear. Addressing this question requires a direct comparison of RAm activation associated with the production of USVs and squeaks.

In the current study, we compared Fos expression in RAm following the production of USVs in adult males and females and following the production of squeaks during courtship interactions (females only). We also directly compared RAm Fos expression associated with the production of USVs and courtship squeaks in females by combining in vivo activity-dependent labeling (TRAP2) with Fos immunohistochemistry in a within-subject design. Our findings refine existing models of premotor control of vocalization and highlight the need to characterize anatomical and functional heterogeneity in vocalization-related RAm neurons.

## Materials and Methods

### Ethics statement

All experiments and procedures were conducted according to protocols approved by the Cornell University Institutional Animal Care and Use Committee (protocol #2020-001).

### Subject details

Male and female TRAP2;Ai14 mice and C57 mice (Jackson Laboratory, 000664) were housed with their siblings and both parents until weaning at Postnatal Day (P)21. TRAP2;Ai14 mice were generated by crossing TRAP2 mice (Jackson Laboratory, 030323) with Ai14 mice (Jackson Laboratory, 007914). All mice were kept on a 12:12 reversed light/dark cycle, were housed in ventilated microisolator cages in a room with regulated temperature and humidity, and were provided with *ad libitum* access to food and water. A running wheel (Innovive) was present in all home cages from the time of weaning and was subsequently removed immediately prior to any behavioral trials that were conducted in the subject mouse's home cage.

### RAm Fos study design

Five groups of C57 mice were subjected to different treatments prior to collecting brains and quantifying Fos expression in RAm: (1) males that produced USVs during courtship interactions with females; (2) females that produced USVs during same-sex interactions with females; (3) females that produced squeaks during courtship interactions with males; (4) males that produced squeaks during a mild footshock paradigm (see below for details of footshock delivery); and (5) females that produced squeaks during a mild footshock paradigm. Subject mice in the male USV, female USV, and female courtship squeak groups were single-housed for 3 d prior to the behavioral trial. The majority of social interaction trials were 30 min in duration, and a subset were shorter (1–10 min in duration) to elicit lower vocalization rates from subject mice. For subjects in the male USV and female USV groups, the subject mouse's home cage was placed in a sound-attenuating recording chamber (Med Associates) equipped with an ultrasonic microphone (Avisoft Bioacoustics), an infrared light source (Tendelux), and a webcam (Logitech, with the infrared filter removed to enable video recording under infrared lighting conditions). A novel, group-housed female was then placed in the home cage of the subject mouse, and vocal and nonvocal behaviors were recorded. For subjects in the female courtship squeak group, the female was placed into the home cage of a novel male inside a sound-attenuating recording chamber, with the bedding removed to facilitate the detection of courtship squeaks. At the end of behavior trials, subject mice remained or were returned to their home cage and were killed 120 min from the start of the behavior trial.

### RAm overlap study design

One group of male TRAP2;Ai14 mice (male USV-USV group) and four groups of female TRAP2;Ai14 mice (female USV-USV, female squeak-squeak, and female squeak-USV, and female USV-squeak groups) were included in this study. Subject mice were single-housed (Day 0) and 3 d later (Day 3) were subjected to social interactions to elicit high rates of either USVs or courtship squeaks (females only). Males and females in the USV-USV groups were given 30-min-long interactions with novel females to elicit high rates of USV production, and females in the squeak-squeak and squeak-USV groups were given 30-min-long courtship interactions with males to elicit the production of high rates of courtship squeaks, as described above. Following these social interactions, subject mice received intraperitoneal injections of 4-hydroxytamoxifen (4-OHT; 50 mg/kg) to enable the expression of tdTomato in recently active neurons, including in RAm neurons that express Fos in association with the production of USVs/squeaks. Ten days later (Day 13), subject mice were given either a 30 min social interaction to elicit USVs (male USV-USV, female USV-USV, and female squeak-USV groups) or a 30 min courtship interaction to elicit squeaks (female squeak-squeak and female USV-squeak groups). At the end of this second behavior trial, subject mice remained or were returned to their home cage and were killed 120 min from the start of the behavior trial.

### Drug preparation

The 4-OHT (HelloBio, HB6040) was dissolved at 20 mg/ml in ethanol by shaking at 37°C and was then aliquoted (75 μl) and stored at −20°C. Before use, 4-OHT was redissolved in ethanol by shaking at 37°C, and filtered corn oil was added (Sigma-Aldrich, C8267, 150 μl). Ethanol was then evaporated by vacuum under centrifugation to give a final concentration of 10 mg/ml, and 4-OHT solution was used on the same day it was prepared, delivered at a final dosage of 50 mg/kg.

### Immunohistochemistry

Two hours following the start of behavior trial, subject mice were deeply anesthetized using isoflurane and then transcardially perfused with phosphate-buffered saline (PBS), pH 7.4, followed by 4% paraformaldehyde (PFA; in 0.1 M PBS), pH 7.4. Brains were subsequently dissected and postfixed in 4% PFA for 24 h at 4°C, followed by immersion in 30% sucrose solution in PBS for 48 h at 4°C. Afterward, brains were embedded in frozen section embedding medium (Surgipath, VWR), flash frozen in a dry ice–ethanol (100%) bath, and then stored at −80°C until sectioning. Coronal sections containing RAm were cut on a cryostat to a thickness of 80 μm, washed in PBS (three times for 5 mins at RT), permeabilized for 2–3 h in PBS containing 1% Triton X-100 (PBST), and then blocked in 0.3% PBST containing 10% Blocking One (Nacalai) for 1 h at RT on a shaker. Sections were then incubated for 24 h at 4°C with primary antibody in blocking solution (1:1,000 rabbit anti-Fos, Cell Signaling Technologies, 2250S), washed three times for 10 min in 0.3% PBST, and then incubated for 24 h at 4°C with secondary antibody in blocking solution (1:1,000, Alexa Fluor 488 goat anti-rabbit, Invitrogen, plus 1:500 NeuroTrace, Invitrogen). Finally, sections were washed two times for 10 min in 0.3% PBST, followed by washing two times for 10 min in PBS. After mounting on slides, sections were dried and coverslipped with Fluromount G (Southern Biotech). Slides were imaged with a 10× objective on a Zeiss 900 laser scanning confocal microscope. To generate the images in Figure 2*A*, immunostaining for Fos was combined with immunostaining for choline acetyltransferase (ChAT; these cases were not used for Fos quantification). In these cases, sections were incubated with primary antibodies in blocking solution (1:1,000 rabbit anti-Fos and 1:1,000 goat anti-ChAT, Millipore, AB144P) and later with secondary antibodies (1:1,000 Alexa Fluor 488 donkey anti-rabbit, Invitrogen, and 1:1,000 Alexa Fluor 594 donkey anti-goat, Invitrogen) but were otherwise processed as described above.

### Confocal image analyses

RAm is located caudal to the nucleus ambiguus in the intermediate reticular formation. In this study, we defined the boundaries of RAm according to the extent of USV-associated Fos expression observed in male mice. For confocal images that included USV-associated RAm Fos and/or tdTomato expression, five coronal sections were selected that included RAm at the following approximate A/P positions: −7.2, −7.4, −7.6, −7.8, and −8.0 mm caudal to the bregma. In each plane of section, oval-shaped regions of interest (ROIs; 200 pixels wide by 400 pixels tall) were generated in ImageJ, rotated 30° (plus 30° for left RAm, minus 30° for right RAm), and then were placed over the bands of Fos-positive and/or tdTomato-positive neurons in the intermediate reticular formation that correspond to RAm. For confocal images from mice that did not include USV-associated RAm Fos or tdTomato, anatomical landmarks (inferior olive, lateral reticular nucleus, spinal trigeminal nucleus) were used to match the placement of RAm ROIs to the placement from images that included USV-associated RAm Fos/tdTomato. For mice in the RAm Fos study, Fos-positive neurons located within these RAm ROIs were manually annotated in each of the five sections by trained observers using ImageJ. For mice in the RAm overlap study, tdTomato-positive neurons located within these RAm ROIs were manually annotated, and overlap was calculated as the proportion of tdTomato-positive RAm neurons that also expressed Fos. Heatmaps of RAm Fos expression and double-labeled RAm neurons (Fos-positive and tdTomato-positive) were constructed as follows. The locations of annotated neurons in each coronal section were first standardized with respect to the position of their RAm ROI (i.e., neuron coordinates were calculated in pixels relative to the centroid of their RAm ROI, with right RAm neuron coordinates flipped horizontally in order to be pooled with the neuron coordinates from left RAm for that same plane of section). To create heatmaps for each plane of section for a single mouse, a bounding rectangle (266 pixels wide by 362 pixels tall) that encompasses the 200 × 400 pixel RAm ROI was created and organized into 13 horizontal bins and 18 vertical bins (each bin ∼20 × 20 pixels). For a given plane of section, the standardized location of each annotated RAm neuron was then placed in its corresponding heatmap bin, and the total number of annotated RAm neurons in each heatmap bin was calculated. This procedure was repeated to generate a heatmap for each plane of section (Sections 1–5) for each mouse. Mean heatmaps for Sections 1–5 were then created for experimental groups by averaging the heatmaps generated for individual mice in that group for each plane of section. Please note that heatmap representations only reflect Fos quantification within the RAm ROIs, and tdTomato-positive and/or Fos-positive neurons outside the RAm ROIs were not counted.

### USV recording and detection

USVs were recorded with an ultrasonic microphone (Avisoft Bioacoustics, CMPA/CM16), amplified (PreSonus TubePreV2), and digitized at 250 kHz (Spike 7, Cambridge Electronic Design). USVs were detected with custom MATLAB codes (Tschida et al., 2019) using the following parameters: mean frequency, >45 kHz; spectral purity, >0.3; spectral discontinuity, <1.0; minimum USV duration, 5 ms; and minimum intersyllable interval, 30 ms.

### Footshock delivery

Subject mice were placed in a footshock chamber (Med Associates) inside a sound-attenuating chamber (Med Associates) equipped with an ultrasonic microphone (Avisoft Bioacoustics) and a webcam (Logitech). A mild (0.5 mA, 1.5 s long) shock was delivered once every 30 s, for a total of 10 footshocks (trial duration, 5 min).

### Squeak detection

In footshock and courtship trials (females only), audio recordings were generated as described above for USV recordings, and a custom MATLAB code was used that allowed trained users to manually annotate the onsets and offsets of individuals squeaks from spectrograms created from audio recordings. The total number of squeaks and the total squeak time were calculated from these annotations.

### Experimental design and statistical analyses

The sample sizes in the five RAm Fos-only groups were as follows: male USV (*n* = 12), female USV (*n* = 16), female courtship squeak (*n* = 20), male footshock (*n* = 4), and female footshock (*n* = 3). The sample sizes in the five RAm overlap (Fos and tdTomato) groups were as follows: male USV-USV (*n* = 5), female USV-USV (*n* = 6), female squeak-squeak (*n* = 6), female squeak-USV (*n* = 5), and female USV-squeak (*n* = 6).

The Shapiro–Wilk test was used to evaluate the normality of data distributions. Parametric, two-sided statistical comparisons were used in all analyses of normally distributed data (one-way or two-way ANOVA, with post hoc Tukey’s HSD when appropriate), and nonparametric, two-sided comparisons were used in analyses that included non-normally distributed data (Mann–Whitney *U* test). In all cases, the significance level was set as *α* = 0.05. No statistical methods were used to predetermine sample size. Errors represent standard error of the mean unless otherwise noted. Statistical analyses were carried out using R 4.4.1 (R Core Team, 2024) and R Studio 2026.01.1 + 403 (Posit team, 2026). Source data generated in this study, as well as custom MATLAB codes, are available upon request.

## Results

### Production of USVs versus courtship squeaks drives different patterns of Fos expression in the RAm

**Figure 1.**
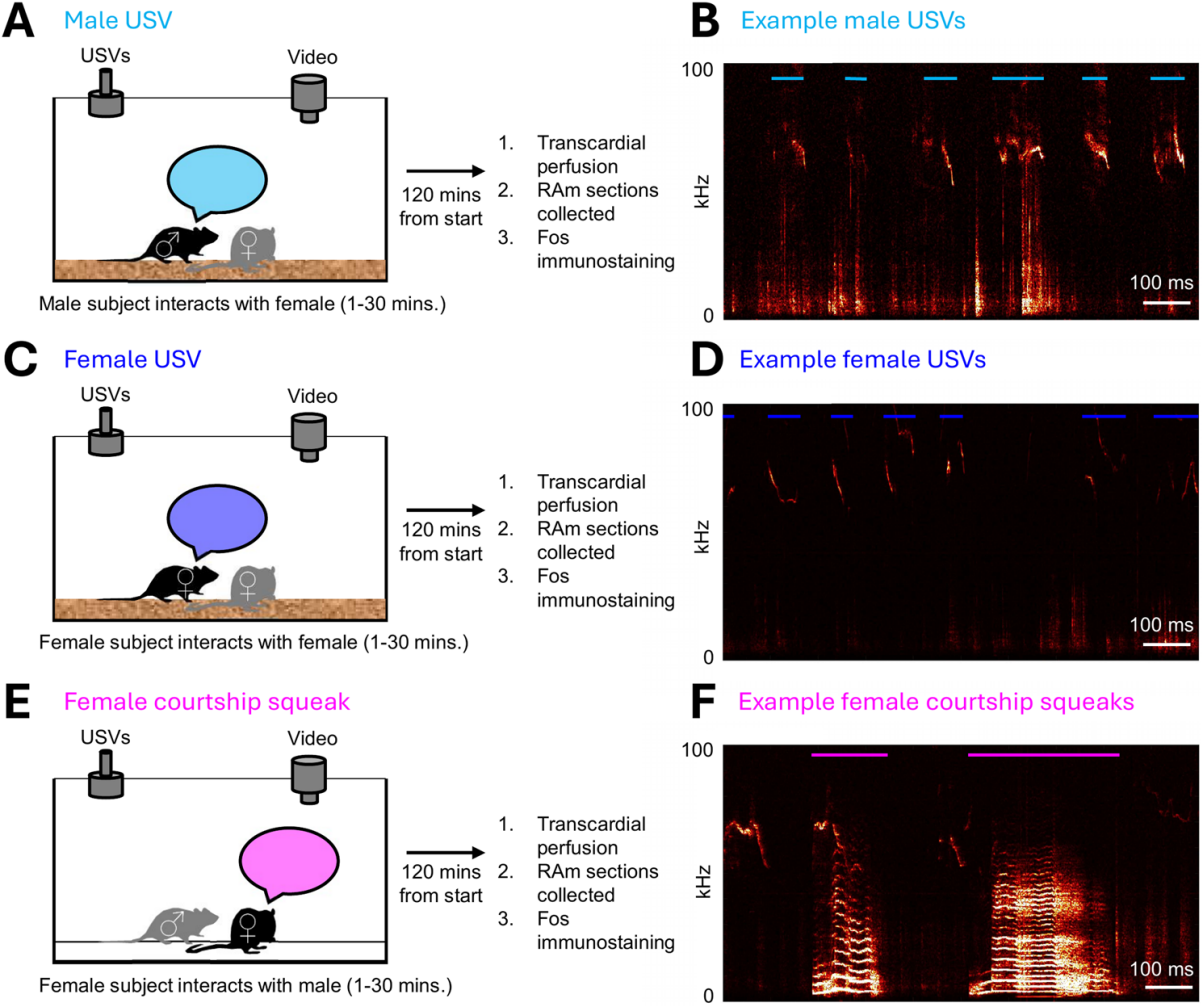
Experimental design of RAm Fos expression experiments. ***A***, Schematic shows procedure for eliciting RAm Fos expression associated with male USV production. ***B***, Spectrogram shows example USVs produced by a male subject. Lines at the top of the spectrogram indicate USV times. ***C***, Schematic shows procedure for eliciting RAm Fos expression associated with female USV production. ***D***, Spectrogram shows example USVs produced by a female subject. Lines at the top of the spectrogram indicate USV times. ***E***, Schematic shows procedure for eliciting RAm Fos expression associated with female courtship squeak production. ***F***, Spectrogram shows example courtship squeaks produced by a female subject. Lines at the top of the spectrogram indicate courtship squeak times. Please note that USVs produced by the stimulus male can also be seen in the spectrogram. Subject mice are shown in black; stimulus mice are shown in gray.

**Figure 2.**
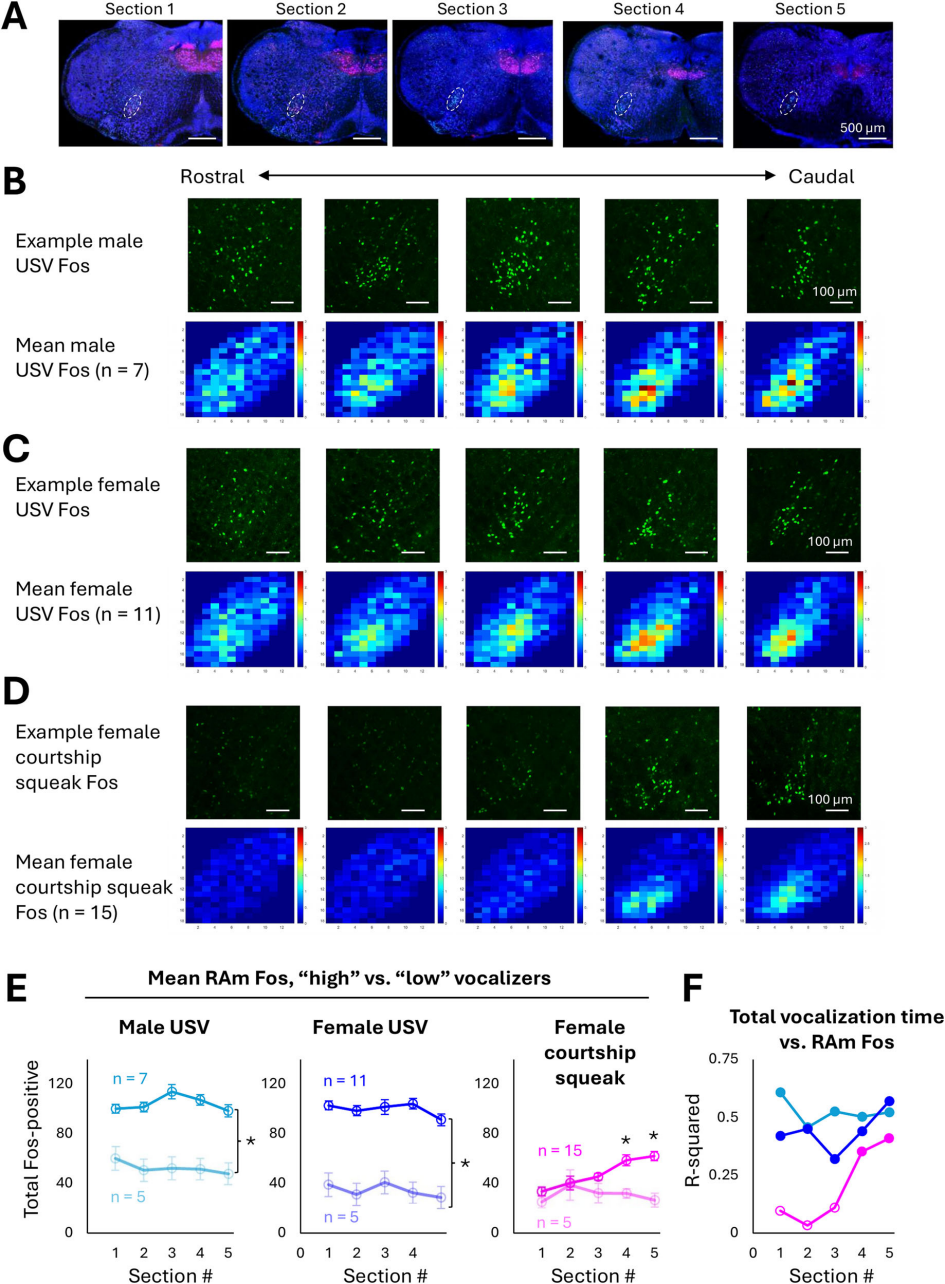
Production of USVs versus squeaks is associated with different patterns of Fos expression in RAm. ***A***, Representative coronal sections show the extent of RAm Fos expression associated with male USV production. White-dotted ovals show ROI placement for Fos scoring. Blue, NeuroTrace; green, Fos; red, ChAT. ***B***, Representative confocal images (top row) show RAm Fos expression in a male that produced USVs during a 30 min courtship interaction with a female. Bottom row shows mean heatmap representations for the high male USV group. ***C***, Same as ***B***, for RAm Fos expression in females that produced USVs during 30 min same-sex interactions with females. ***D***, Same as ***B***, for RAm Fos expression in females that produced courtship squeaks during 30 min interactions with males. ***E***, Mean counts of Fos-positive RAm neurons are shown by section (1–5) in high versus low USV males (left), high versus low USV females (middle), and high versus low courtship squeak females (right). ***F***, Plot shows the *R*^2^ values for the correlation between total Fos-positive RAm neurons and total vocalization time for each group and for each RAm section. Filled points indicate statistically significant relationships, and open points indicate nonsignificant relationships. See also Extended Data Figures 2-1–2-3.

As a first step toward comparing Fos expression in RAm associated with the production of USVs versus squeaks, we characterized the rostral-to-caudal extent of RAm Fos expression associated with USV production in male mice (Fig. 1*A*). Although past studies have reported that RAm neurons upregulate Fos expression in males that recently produced high rates of courtship USVs (Tschida et al., 2019; Concha-Miranda et al., 2022; Veerakumar et al., 2023; Park et al., 2024), the spatial extent of USV-associated RAm Fos expression has not been described. To this end, male subjects were single-housed for 3 d to promote high rates of USV production and then were given 30 min social interactions with novel, group-housed females. Males were perfused 120 min later, and hindbrain sections were collected and stained for Fos expression, starting at the level of the nucleus ambiguus and extending caudally to the hindbrain–spinal cord transition. We found that male USV production is associated with robust Fos expression in neurons within the intermediate reticular formation, beginning around −7.2 mm caudal to bregma and extending caudally ∼1 mm (*n* = 7 high USV males; Fig. 2*A*,*B*,*E*; see Materials and Methods for details of heatmap representations). To better evaluate the relationship between RAm Fos expression and male USV production, we also added behavioral data and measured RAm Fos expression from *n* = 5 “low USV” males that were given brief (1–10 min) social interactions in order to elicit lower USV rates. High versus low USV males differed significantly in the total numbers of Fos-positive neurons across all five RAm sections (Fig. 2*E*; *F*_(1,10)_ = 17.03; *p* = 0.002 for main effect of group; *p* > 0.05 for main effect of section and for interaction effect; two-way ANOVA with repeated measures on one factor). When we pooled high and low male USV cases to calculate the correlation between total vocalization (USV) time and total Fos-positive neurons for each RAm section (1–5), we found that total USV time was significantly correlated with RAm Fos expression across all five RAm sections in the male USV group (Fig. 2*F*; Extended Data Fig. 2-1; *p* < 0.05 for all male USV comparisons; linear regressions; see Extended Data Fig. 2-2 for correlations in high vs low USV male groups). We subsequently defined the boundaries of RAm according to this male USV-associated pattern of Fos expression (see Materials and Methods).

10.1523/ENEURO.0100-26.2026.f2-1 Figure 2-1 **Relationship between total vocalization time and total Fos-positive RAm neurons.** These plots show the relationship between total vocalization time (in seconds; either USVs or courtship squeaks) vs. total Fos-positive RAm positive neurons, by RAm section (1-5) for each experimental group. Light blue, male USV (n = 12); dark blue, female USV (n = 16); magenta, female courtship squeak group (n = 20). R-squared values and p values for statistically significant relationships are shown in bold font. Download Figure 2-1, TIF file.

10.1523/ENEURO.0100-26.2026.f2-2 Figure 2-2 **Relationship between total vocalization time and total Fos-positive RAm neurons for long (30-minute) vs. short (<5-minute) duration trials.** These plots show the relationship between total vocalization time (in seconds; either USVs or courtship squeaks) vs. total Fos-positive RAm positive neurons, by RAm section for each experimental group. Total Fos-positive RAm neurons was calculated across sections 1-5 for the USV groups and across sections 4-5 for the female courtship squeak group. Row 1 shows data from male USV group, row 2 shows data from female USV group, and row 3 shows data from female courtship squeak group. Left column plots show relationships including all trials, and right column plots show relationships separately for long duration trials (blue points) and short duration trials (orange points). R-squared values and p values for statistically significant relationships are shown in bold font. Download Figure 2-2, TIF file.

We next asked whether USV production in female mice was associated with a similar pattern of RAm Fos expression (Fig. 1*B*). Past work has shown that females produce low-to-negligible rates of USVs during courtship interactions with males (Whitney et al., 1973; Warburton et al., 1989; White et al., 1998; Neunuebel et al., 2015; Heckman et al., 2017; Matsumoto et al., 2022; Oliveira-Stahl et al., 2023; Sterling et al., 2023; Malone et al., 2025; Waidmann et al., 2025). To elicit robust USV production from females, 3 d single-housed female subjects were given 30 min social interactions with novel females. Although we did not employ any method to assign individual USVs to the subject female versus the novel female (Warren et al., 2020), past work found that short-term social isolation promotes multiple aspects of female social behavior during same-sex interactions, including USV production (Zhao et al., 2021, 2025). Moreover, the observed high USV rates suggest that single-housed subject females vocalize robustly during these interactions (mean total female–female USVs = 2567 ± 292). Correspondingly, female subjects exhibited robust RAm Fos expression with the same localization seen in males (*n* = 11 high USV females; Fig. 2*C*). To evaluate the relationship between RAm Fos expression and female USV production, we added behavioral data and measured RAm Fos expression from *n* = 5 “low USV” females that were given brief social interactions in order to elicit lower USV rates. Similar to the male USV group, high versus low USV females differed significantly in the total numbers of Fos-positive neurons across all five RAm sections (Fig. 2*E*; *F*_(1,14)_ = 85.36; *p* < 0.001 for main effect of group; *p* > 0.05 for main effect of section and for interaction effect; two-way ANOVA with repeated measures on one factor). Also like the male USV group, when we considered the correlation between total vocalization (USV) time and total Fos-positive neurons for each RAm section (1–5), we found that total USV time was significantly correlated with RAm Fos expression across all five RAm sections in the female USV group (Fig. 2*F*; Extended Data Fig. 2-1; *p* < 0.05 for all female USV comparisons; linear regressions; see Extended Data Fig. 2-2 for correlations in high vs low USV female groups). In summary, USV production is associated with robust and similar patterns of RAm Fos expression in males and females.

Having characterized the spatial extent of RAm Fos expression associated with USV production, we next characterized RAm Fos expression associated with the production of squeaks. We initially considered RAm Fos expression in a small number of male and female mice that were subjected to a mild footshock paradigm to elicit squeaks (Extended Data Fig. 2-3; see Materials and Methods; *n* = 3 female footshock; *n* = 4 male footshock). However, these mice produced only modest rates of squeaks (20–60 total squeaks), and the total number of Fos-positive RAm neurons was low relative to females and males that produced high rates of USVs (Extended Data Fig. 2-3; mean total RAm Fos-positive neurons: male footshock = 93 ± 21; female footshock = 193 ± 29; female USV = 499 ± 15; male USV = 545 ± 36). In male mice, we are unaware of an alternative behavioral paradigm that would enable us to elicit substantially higher squeak rates without also risking injury to our subject animals (e.g., interactions with an aggressive male). However, past studies have recorded high rates of squeaks during courtship interactions between female and male mice, and these studies also provide evidence that females produce the majority of squeaks during these interactions (Wang et al., 2008; Grimsley et al., 2013: Finton et al., 2017; Klaus et al., 2025). Therefore, as an alternative paradigm to elicit higher rates of squeaks in females, we measured female squeak rates during courtship interactions with males (i.e., courtship squeaks; Fig. 1*C*). We found that females produced robust rates of squeaks during 30 min courtship interactions with sexually experienced males (Extended Data Fig. 2-3*C*; mean total courtship squeaks = 233 ± 31; *t*_(20)_ = 3.85; *p* < 0.001 for difference in total squeaks between courtship and footshock groups; *t* test). When we examined RAm Fos expression in female mice that produced high rates of courtship squeaks, we found robust Fos expression, but in a pattern that differed in two ways from RAm Fos expression associated with USV production. First, courtship squeak production is associated with overall weaker RAm Fos expression than either male or female USV production (Fig. 2*D*; *F*_(2,30)_ = 71.31; *p* < 0.001 for main effect of group; *p* < 0.0001 for male USV vs female courtship squeak; *p* < 0.0001 for female USV vs female courtship squeak; one-way ANOVA plus post hoc Tukey's HSD). Second, we also observed a difference in the localization of squeak-associated RAm Fos expression. Whereas USV production was associated with robust Fos expression across all five representative planes of section, squeak production was associated with robust Fos expression in only the caudal-most RAm sections (Sections 4 and 5; Fig. 2*D*). To evaluate the relationship between this pattern of RAm Fos expression and female courtship squeak production, we added behavioral data and measured RAm Fos expression from *n* = 5 “low courtship squeak” females that were given brief social interactions with males in order to elicit lower squeak rates. Notably, the mean total counts of Fos-positive RAm neurons tended to be higher in RAm Sections 4 and 5 but differed significantly only in the caudal-most RAm between high courtship squeak and low courtship squeak females (Fig. 2*E*; *F*_(2,32)_ = 5.0; *p* = 0.008 for interaction between group and section; *p* = 0.004 for Section 5 high squeak vs low squeak; two-way ANOVA with repeated measures on one factor plus post hoc Tukey's HSD for across-group, within-section comparisons). Similarly, when we considered the correlation between total vocalization (squeak) time and RAm Fos expression for each RAm section, we found that total squeak time was only significantly correlated with RAm Fos expression in Sections 4 and 5 (Fig. 2*F*; Extended Data Fig. 2-1; *p* < 0.05 for Section 4 and section 5 comparisons only; linear regressions; see Extended Data Fig. 2-2 for correlations in high vs low courtship squeak female groups). In summary, these data show that production of female/male USVs and female courtship squeaks are associated with different patterns of Fos expression in RAm.

**Figure 3.**
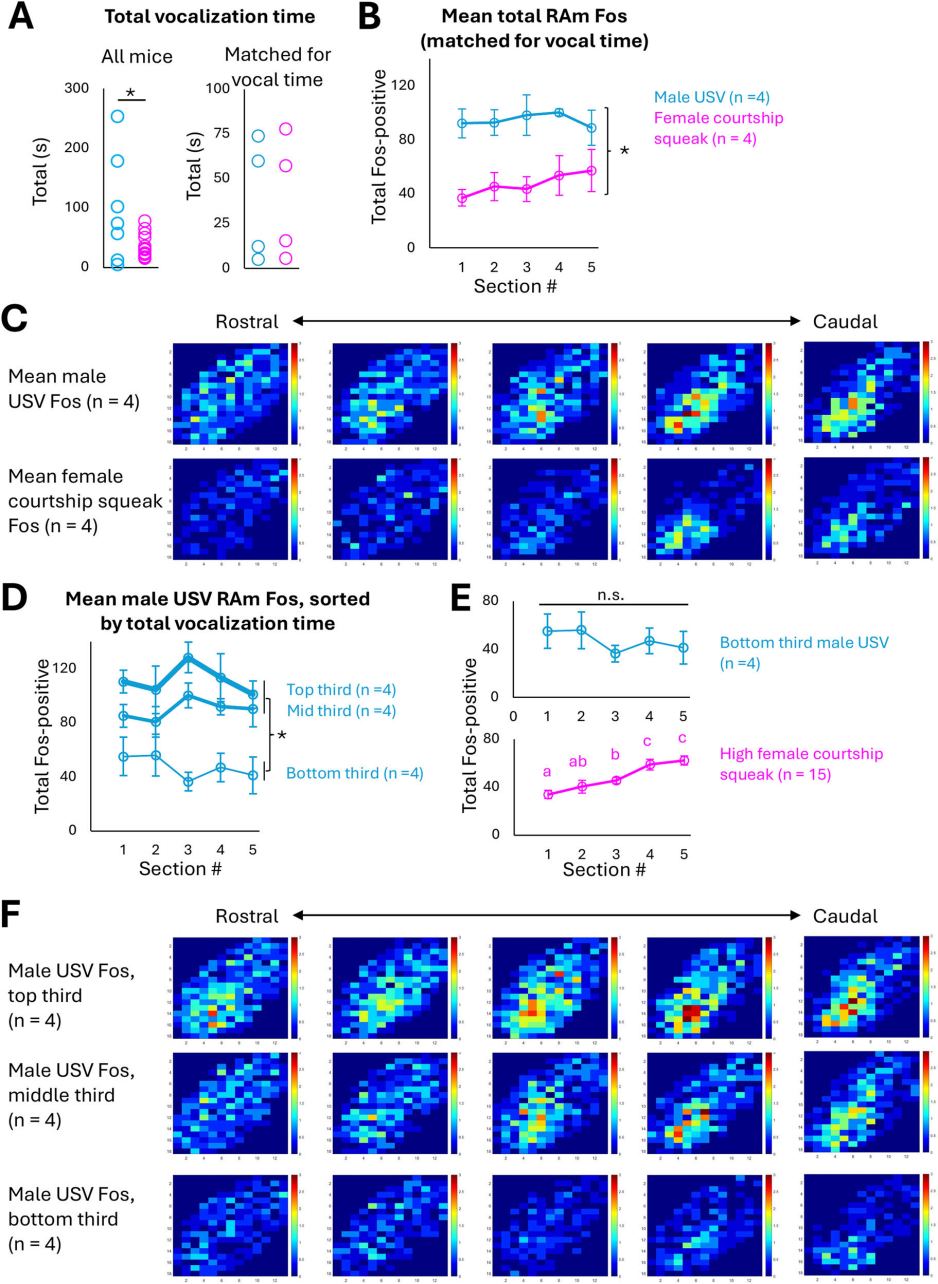
Differences in RAm Fos expression following the production of USVs versus courtship squeaks are not accounted for by differences in total vocalization time. ***A***, Left, Total vocalization time is plotted for all high male USV cases (*n* = 7, blue) and high female courtship squeak cases (*n* = 15, magenta). Right, Total vocalization time is plotted for *n* = 4 high male USVs cases and *n* = 4 high female courtship squeak cases that are matched for vocalization time. ***B***, Mean counts of Fos-positive RAm neurons are plotted by section for the subset of male USV and courtship squeak cases matched for total vocalization time. ***C***, Heatmaps show mean RAm Fos expression by section for the subset of male USV and courtship squeak cases matched for total vocalization time. ***D***, Mean counts of Fos-positive RAm neurons are plotted by section for male USV cases split into groups according to total vocalization time. ***E***, Mean counts of Fos-positive RAm neurons are plotted by section for the bottom-third male USV cases (*n* = 4, top) and for the high female courtship squeaks cases (*n* = 15, bottom; replotted from Fig. 2*E*). ***F***, Heatmaps show mean RAm Fos expression by section for the top-third, middle-third, and bottom-third of male USV cases, grouped by total vocalization time.

10.1523/ENEURO.0100-26.2026.f2-3 Figure 2-3 **Comparison of squeak production and RAm Fos expression in the female courtship and male/female footshock groups.** (A) Heatmap representations show mean RAm Fos expression in males and females exposed to a mild footshock paradigm. (B) Mean counts of Fos-positive RAm neurons are shown by section (1-5) in females that produced courtship squeaks (magenta, n = 15), females that received footshocks (black, n = 3), and males that received footshocks (gray, n = 4). Different lowercase letters indicate statistically significant differences between groups for a given RAm section (F(4,46) = 3.32, p = 0.016 for interaction between group and section; two-way ANOVA with repeated measures on one factor plus Tukey’s posthoc for within-section, across-group comparisons only). (C) Females produce significantly greater counts of squeaks during 30-minute courtship interactions with males than do males/females exposed to a mild footshock paradigm (t(20) = 3.85, p < 0.001; unpaired t-test). Download Figure 2-3, TIF file.

One possibility is that the difference in counts and spatial distributions of Fos-positive RAm neurons associated with USVs versus squeaks is related to the production of different vocalization types per se. However, another possibility is that the difference in RAm Fos expression is related to the difference in total vocalization time between the USV and courtship squeak groups. Although we were able to elicit robust squeak rates using the female courtship squeak paradigm, females nonetheless spent on average less total time squeaking than males spent producing USVs (Fig. 3*A*; *z* = 3.67; *p* < 0.001; Mann–Whitney *U* test; female USV group not included in comparison because the number of USVs produced by subject females versus novel females was not determined). To test whether a difference in vocalization time could account for the observed difference in RAm Fos expression patterns, we conducted two analyses. First, we selected a subset (*n* = 4) of male USV cases that were well matched in their total vocalization time to a subset (*n* = 4) of female courtship squeak cases (Fig. 3*A*). A comparison of these cases showed that male USV production was still associated with greater number of Fos-positive RAm neurons than female courtship squeak production (Fig. 3*B*; *F*_(1,6)_ = 13.28; *p* = 0.01 for main effect of group; *p* > 0.05 for main effect of section and for interaction effect; two-way ANOVA with repeated measures on one factor). Moreover, heatmap representations of the mean RAm Fos expression for the two groups revealed patterns of Fos expression that resemble Fos expression in the full male USV and full female courtship squeak groups (compare Figs. 3*C*, 2*C*,*D*).

Second, we split the male USV cases into three groups according to their total vocalization time (Fig. 3*D–F*; *n* = 4 males in the top-third, *n* = 4 males in the middle-third, and *n* = 4 males in the bottom-third USV groups). Total RAm Fos expression was higher in the top-third and middle-third male USV groups than in the bottom-third male USV group (Fig. 3*D*; *F*_(2,9)_ = 10.17; *p* = 0.005 for main effect of group; *p* > 0.05 for main effect of section and for interaction effect; *p* = 0.004 for top vs bottom; *p* = 0.04 for mid vs bottom; two-way ANOVA with repeated measures on one factor plus post hoc Tukey's HSD). Even in the bottom-third male USV group, however, we found that the distribution of Fos-positive RAm neurons across Sections 1–5 still looked “USV-like” rather than “squeak-like.” That is to say, in the high female courtship squeak group, RAm Fos expression is significantly higher in the caudal sections of RAm than in the rostral sections of RAm (Fig. 3*E*; magenta points show high female courtship squeak data replotted from Fig. 2*E*; *F*_(2.16,30.23)_ = 14.40; *p* < 0.001 for main effect of section; one-way ANOVA with repeated measures plus post hoc Tukey’s HSD; different lowercase letters indicate significant differences between sections). In contrast, in the bottom-third male USV group, RAm Fos expression is not significantly different between the rostral and caudal sections of RAm (Fig. 3*E*; *F*_(4,12)_ = 1.59; *p* > 0.05 for main effect of section; one-way ANOVA with repeated measures). Heatmap representations of mean RAm Fos expression also show that although RAm Fos expression is weakest in the bottom-third male USV group, Fos expression remains distributed across both rostral and caudal RAm sections (Fig. 3*F*). In summary, these analyses indicate that differences in total vocalization time cannot account for the different patterns of RAm Fos expression associated with the production of USVs and courtship squeaks. Rather, these differences support the conclusion that at least somewhat distinct sets of RAm neurons upregulate Fos in association with the production of USVs versus squeaks.

### Only a subset of RAm neurons express Fos in association with the production of both USVs and squeaks

**Figure 4.**
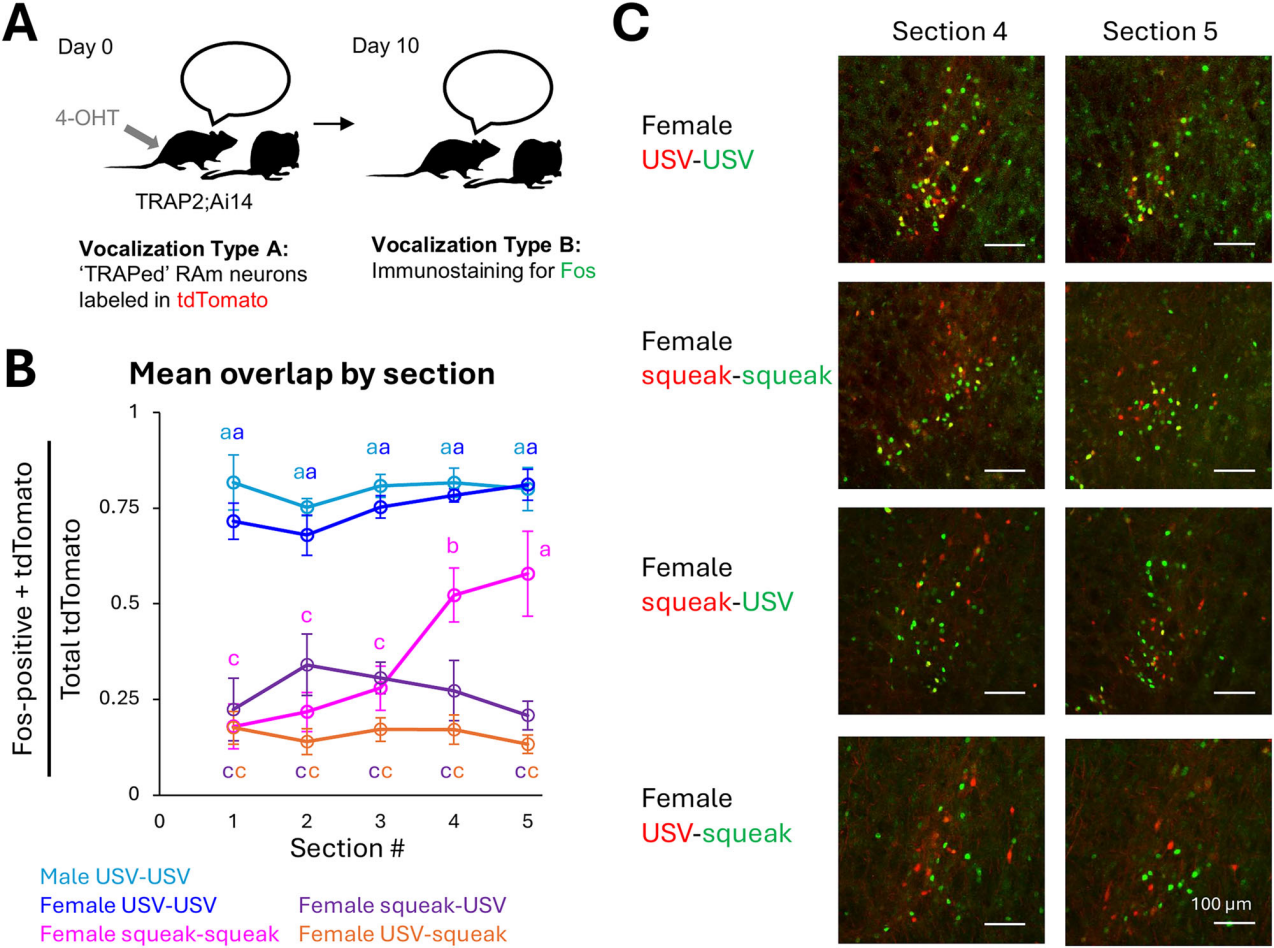
Only a subset of RAm neurons express Fos in association with the production of both USVs and squeaks. ***A***, Experimental design. ***B***, Mean overlap (proportion of tdTomato RAm neurons that are also Fos-positive) is plotted for each group and for each RAm section. Different lowercase letters indicate statistically significant differences between groups for a given RAm section. ***C***, Representative confocal images showing RAm neurons labeled with tdTomato following behavior 1 (USVs or courtship squeaks) and RAm neurons that are Fos-positive following behavior 2 (USVs or courtship squeaks). Red, tdTomato; green, Fos. See also Extended Data Figure 4-1.

The Fos datasets indicate that squeak production is associated with Fos expression biased toward caudal RAm, while USV production is associated with broader Fos expression throughout rostral and caudal RAm. To what degree are RAm neurons recruited in association with both vocalization types? On the one hand, RAm neurons that are activated in association with squeak production could represent a nested subset of the RAm neurons that are activated in association with USV production, similar to the organization recently described for ventral nerve cord circuits that regulate the production of pulse and sine courtship song in *Drosophila* males (Lillvis et al., 2024; Shiozaki et al., 2024). Alternatively, RAm might contain USV-related neurons, squeak-related neurons, and shared neurons that are recruited during both vocalization types. To directly compare Fos expression associated with the production of USVs versus squeaks in the same animals, we combined in vivo activity-dependent labeling (TRAP2) with Fos immunohistochemistry in a within-subject design (Fig. 4*A*). Briefly, each TRAP2;Ai14 subject was exposed to a behavioral context that would elicit production of high rates of USVs (males/females) or high rates of courtship squeaks (females only) and then was treated with 4-OHT to drive tdTomato expression in neurons that upregulated Fos in association with that vocalization type, including RAm neurons. Ten days later, subjects were either placed in the same context a second time or were placed in the alternate context (females only). Two hours after this second session, brains were collected, and RAm sections were immunostained for Fos expression.

10.1523/ENEURO.0100-26.2026.f4-1 Figure 4-1 **Heatmap representations of double-labeled RAm neurons (tdTomato-positive and Fos-positive) from RAm overlap experiments.** Heatmaps show the mean distribution of double-labeled RAm neurons. Each row shows a different experimental group, and columns show RAm sections 1-5. Download Figure 4-1, TIF file.

We first evaluated control datasets (*n* = 5 male USV-USV, *n* = 6 female USV-USV, and *n* = 6 female squeak-squeak mice) to ensure that the TRAP2 strategy permits reliable labeling of both USV-related and squeak-related RAm neurons. In the male and female USV-USV groups, a large proportion of tdTomato-expressing RAm neurons that were labeled following the first round of USV production were Fos-positive after the second round of USV production, and mean overlap was high across both rostral and caudal RAm sections (Fig. 4*B*,*C*; mean overlap across Sections 1–5 for the male USV-USV group is 79.9 ± 8.6%; mean overlap across Sections 1–5 for the female USV-USV group is 74.8 ± 6.1%). Heatmap representations of the double-labeled RAm neurons in the female and male USV-USV groups also resemble the pattern of RAm Fos expression following USV production (compare Extended Data Fig. 4-1 with Fig. 2*B*,*C*), indicating that TRAP2 can efficiently label USV-related RAm neurons. Given that Fos expression associated with squeak production is highest in caudal RAm (Fig. 2*D*,*E*) and is only significantly correlated with total squeak time in RAm Sections 4 and 5 (Fig. 2*F*), we anticipated that the overlap between tdTomato and Fos in the female squeak-squeak group would be highest in caudal RAm. In line with this prediction, we found that mean overlap in the female squeak-squeak group was highest in RAm Sections 4 and 5 (Fig. 4*B*,*C*), and heatmap representations of the double-labeled RAm neurons in the squeak-squeak group resemble the pattern of RAm Fos expression following courtship squeak production (compare Extended Data Fig. 4-1 with Fig. 2*D*). We note that the mean overlap in Sections 4 and 5 is lower in the female squeak-squeak group than in the USV-USV groups, possibly because squeak-associated RAm Fos expression is weaker than USV-associated Fos expression (mean overlap across Sections 4 and 5: female squeak-squeak = 54.6 ± 19.6%; male USV-USV = 81.1 ± 9.5%; female USV-USV = 79.9 ± 6.2%). Nonetheless, these data indicate that TRAP2 can reliably label both USV-related and squeak-related ensembles of RAm neurons.

**Figure 5.**
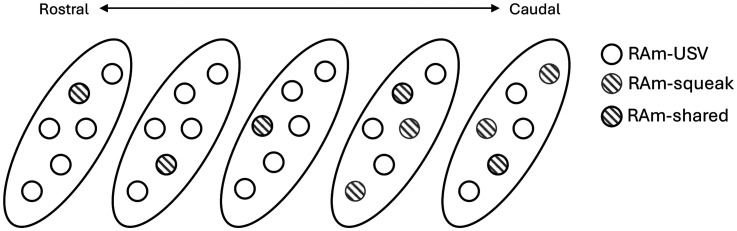
Working model of RAm organization with respect to the vocalization type. Our data suggest that RAm may contain USV-related neurons, squeak-related neurons, and shared neurons that are recruited during the production of both vocalization types.

We next tested whether the same RAm neurons are Fos-positive in association with the production of both vocalization types by evaluating overlap in the female squeak-USV group (*n* = 5) and the female USV-squeak group (*n* = 6). Notably, overlap in these experimental groups was significantly lower across all RAm sections than the USV-USV control groups and was also lower across caudal RAm sections than the female squeak-squeak control group (Fig. 4*C*; *F*_(10.76, 61.85)_ = 4.91; *p* < 0.001 for interaction effect; one-way ANOVA plus post hoc Tukey’s HSD for within-section, across-group comparisons only; for each RAm section, significant differences between groups are indicated by different lowercase letters). Together with our Fos datasets, these findings do not support the idea that squeak-related RAm neurons comprise a subset of RAm neurons recruited during USV production. Rather, they suggest a model in which there are at least three types of vocalization-related RAm neurons: (1) RAm-USV neurons, which are recruited during USV production and are distributed throughout rostral and caudal RAm; (2) RAm-squeak neurons, which are recruited during squeak production and are localized in caudal RAm; and (3) RAm-shared neurons, which are recruited during both vocalization types and may be distributed throughout rostral and caudal RAm (Fig. 5).

## Discussion

In the current study, we compared patterns of Fos expression in RAm associated with production of USVs by female and male mice and production of courtship squeaks by females. Our across-subjects Fos datasets provide evidence that USV production is associated with robust Fos expression in both rostral and caudal RAm, whereas female courtship squeak production is associated with overall weaker Fos expression that is biased toward caudal RAm. Control comparisons indicate that these differences in RAm Fos expression are related to the production of different vocalization types rather than by a difference in time spent vocalizing between the USV and courtship squeak groups. Finally, our within-subjects overlap dataset reveals that only a subset of RAm neurons are Fos-positive following the production of both vocalization types, which suggests that the majority of vocalization-related RAm neurons are not recruited in a shared manner across both vocalization types but rather are recruited selectively during either the production of USVs or squeaks.

Our finding that squeak production is associated with fewer Fos-positive RAm neurons than USV production intersects with recent work characterizing the role of *Nts*-expressing RAm neurons in mouse vocal production (Veerakumar et al., 2023). Specifically, Veerakumar et al. found that low-frequency optogenetic activation of RAm *Nts* neurons elicits squeak-like (< 20 kHz) vocalizations, while higher-frequency optogenetic activation (which presumably drives stronger activation of individual RAm neurons, as well as the activation of greater numbers of RAm neurons) drives the production of vocalizations in the ultrasonic range. These findings are at face value concordant with our observation that USV production is associated with more robust and more spatially extensive RAm Fos expression than courtship squeak production. Nonetheless, it is somewhat counterintuitive that natural squeak production recruits fewer Fos-positive RAm neurons than the production of USVs. On the one hand, RAm has been implicated in regulating expiratory force and, in turn, vocalization amplitude (i.e., loudness; Jürgens, 2002; Concha-Miranda et al., 2022; Veerakumar et al., 2023), and so one idea is that increasing RAm activation drives increasing expiratory force in a monotonic fashion and therefore leads to a switch from voiced (i.e., human-audible) vocalizations to USVs. However, past work in rats reported that subglottal pressure is lower during the production of USVs than during the production of squeaks (Riede, 2018). Together with our data showing that only a subset of RAm neurons are Fos-positive following the production of both vocalization types, these findings are difficult to reconcile with the idea that RAm vocalization-related neurons comprise a homogenous population, with increasing activation driving increased expiratory force and therefore a switch from voiced vocalizations to USVs. Given that USVs and squeaks are produced via distinct sound production mechanisms, via vibration of the vocal folds to generate squeaks and via an intralaryngeal whistle mechanism to generate USVs (Mahrt et al., 2016; Riede et al., 2017; Håkansson et al., 2022), a different possibility is that largely nonoverlapping sets of RAm neurons are recruited during the production of voiced vocalizations versus USVs.

We hypothesize that Fos-positive RAm neurons associated with the production of USVs versus squeaks may differ substantially in their connectivity with vocal–respiratory hindbrain neurons. Because the production of rodent USVs relies predominantly on laryngeal but not articulatory mechanisms (Mahrt et al., 2016; Riede et al., 2017; Håkansson et al., 2022), one possibility is that the axonal projections of USV-related RAm neurons are biased toward nucleus ambiguus and spinal expiratory centers, while squeak-related RAm neurons may have particularly strong projections to expiratory centers, in addition to nucleus ambiguus and brainstem articulatory motor neurons. It is also possible that activation of USV-related and squeak-related RAm neurons may recruit different subsets of motor neurons within a given motor nucleus. In mammals, RAm has been shown to innervate the spinal cord expiratory centers, nucleus ambiguus, respiration-related hindbrain regions, as well as the trigeminal, facial, and hypoglossal nuclei (Holstege, 1989; Veerakumar et al., 2023). In the zebra finch, RAm neurons that send axonal projections to vocal motor neurons in the hypoglossal nucleus (which in turn innervate the bird’s vocal organ, the syrinx) are spatially intermingled with but distinct from RAm neurons that project to expiratory-related neurons in the thoracic spinal cord (Wild et al., 2009). However, the extent to which projection-defined RAm populations in the mouse are distinct, overlapping, and/or organized topographically remains poorly described. Our findings highlight the need to characterize heterogeneity within vocalization-related RAm neurons and to further characterize our Fos-identified populations in terms of their inputs, axonal projections, and expression of molecular markers. Such advances would in turn facilitate the study of these vocalization-related RAm neurons in both females and males.

We also note that there is past work in rats suggesting that RAm neurons differentially regulate the production of different vocalizations. Hartmann and Brecht found that microstimulation of RAm elicited the production of low-frequency (< 30 kHz) USVs but not high-frequency (> 30 kHz) USVs, and local cooling of RAm blocked the production of low-frequency USVs elicited by PAG stimulation but did not block PAG-elicited high–frequency USVs (Hartmann and Brecht, 2020). Although that study did not evaluate the role of RAm in regulating the production of squeaks, their findings provide additional evidence that RAm neurons participate differentially in the production of different vocalization types.

Past work also explored whether distinct or overlapping populations of PAG neurons regulate the production of different vocalization types. Older work found that partial PAG lesions abolish the production of some vocalization types while leaving others intact, suggesting that different populations of PAG neurons may control the production of different vocalization types (Kelly et al., 1946; Skultety, 1962; Newman and MacLean, 1982; Jürgens, 1994). In recent work from our group, we used TRAP2 activity-dependent labeling to express caspase in PAG neurons whose activity is critical for the production of USVs. This manipulation blocks the production of USVs in both male and female mice but spares the production of squeaks (including footshock-elicited squeaks in males and females, as well as female courtship squeaks), suggesting that distinct PAG neurons regulate the production of USVs versus squeaks (Ziobro et al., 2024). The findings of the current study refine our understanding of how regions downstream of the PAG are organized to regulate the production of different vocalization types and suggest that even in the hindbrain, vocalization-related premotor neurons recruited during the production of USVs and squeaks may be largely nonoverlapping.

An important limitation of the current study is that the absence of Fos expression does not correspond to the absence of neural activity. Although our results support the conclusion that a larger and more spatially distributed population of RAm neurons is strongly recruited during the production of USVs than during the production of squeaks, our results do not rule out the possibility that some RAm neurons exhibit activity during the production of both vocalization types that fails to drive Fos expression. In vivo electrophysiological measurements and functional manipulations of neural activity in these Fos-defined populations, as well as more generally in rostral versus caudal RAm neurons, will be important to further characterize these neurons and their contributions to vocal production. Our findings form a foundation to understand how hindbrain circuits are organized to regulate the production of different vocalization types, including in future work that could explore how vocalization-related neurons in RAm and other hindbrain regions are organized to regulate vocal production in different species with diverse vocal repertoires.

## Synthesis

Reviewing Editor: Charlotte Cornil, Universite de Liege Decisions are customarily a result of the Reviewing Editor and the peer reviewers coming together and discussing their recommendations until a consensus is reached. When revisions are invited, a fact-based synthesis statement explaining their decision and outlining what is needed to prepare a revision will be listed below. The following reviewer(s) agreed to reveal their identity: J Martin Wild, Arkarup Banerjee. Note: If this manuscript was transferred from JNeurosci and a decision was made to accept the manuscript without peer review, a synthesis may not be available.

Your manuscript has now been reviewed by two experts who agree on the importance of identifying specific populations of RAm premotor neurons involved vocalizations and on the quality of rostro-caudal mapping of neurons that cFos neurons following production of USVs or squeaks as well as the approach to determine the spatial distribution of neurons active during functionally distinct vocalizations within a same individual. They also praise the quality of the writing which contains clearly stated objectives, a nice summary of the existing relevant data and clearly described methodology and provides relevant answers to the questions that are raised. However, they raise a few concerns that require to be addressed for this manuscript to be considered for publication. These concerns are detailed here below with specific recommendations to address them.

Major concerns:

1. Due to the inherent limitations of cFos, a technique which identifies neural activity correlated with behavior, one cannot conclude that the cFos activated neurons drive squeaks or USVs. In order to claim that RAm contains three vocalization-related populations of neurons, the authors would ideally need to conduct perturbation experiments, for example, by combining TRAP2 experiments with loss of function perturbations (e.g., TeLC) demonstrating causal contributions.

Alternatively, in the absence of causal demonstration, the authors should adjust the language in the paper to reflect that these neurons are correlated/associated with the two vocal modes and avoid language that conflates correlation with causation. For example, in the second sentence of discussion (line number 475), the authors write that "USV production drives robust cFos expression". Similar language is used in multiple places in the results and discussion sections. It is recommended that these be adjusted to simply say that "cFos expression is correlated with USV production", in the absence of additional functional experiments.

2. Do we know that all squeaks are emitted by female mice? This is a critical assumption underlying the experimental design. We understand that male mice produce the majority of USVs in a courtship context but could the authors cite relevant literature (e.g., in line 350) pointing out that courtship squeaks are produced predominately by females and not by males?

3. The rationale for using female-female interactions to elicit USVs in females rather than male-female interactions should be clarified. Should we assume that all USVs regardless of the context in which they are produced are associated with similar cFos expression in RAm?

4. While manipulating interaction time to assign high-vocalizer vs. low-vocalizer groups is a reasonable strategy, it may also conflate with other non-vocal behaviors (such as locomotion, licking, grooming, etc) that may scale with interaction time. So, our concern is that neurons identified in Fig 1E may be involved in other non-vocal behaviors spuriously correlated with interaction time. Can the authors resolve this issue? One possibility would be to leverage the natural variability in vocalizations rates across animals. Could you show that the total number of cFos positive neurons scales with the number of vocalizations across animals that are allowed to interact for the same amount of time?

Minor comments:

1. While the authors mention the difference in functional context of squeaks vs. USVs in the introduction, a discussion on the mechanism of vocal production seems to be missing. USVs are produced by a whistle mechanism whereas squeaks are produced by vibration of the vocal cords. Could the authors clarify/speculate on how their result of differential recruitment of RAm neurons constrain the sound production mechanism?

2. lines 54-55 seem to suggest that the different sets of muscles involved in vocalisation "overlap", which is clearly not the case: the differently involved RAm neurons may overlap, but the muscles do not!

3. Please define "RAm voc neurons". Although this may seem intuitive it may not be for all readers.

4. For Fig. 3b, could the authors compare the observed proportion of squeak neurons that are also USV neurons (double positive) against the expected proportion if these two populations were simply independent. Under the independence model, the expected probability of double positive neurons is simply the product of the two individual probabilities: P(squeak)* P(USV) = P (squeak ∩ USV). This would provide quantitative evidence of whether the proportion of double positive neurons is simply expected by chance, consistent with their hypothesis of USVs and Squeak recruiting independent neuronal populations in the RAm.

5. In line 493 'that' should be 'than' since a comparison is involved.

## Author Response

We thank the Reviewers and the Editor for their thoughtful and helpful comments. We believe that our manuscript has improved after addressing these concerns. Below, we address in turn the specific concerns raised by each Reviewer. Please note that we have also made minor formatting changes to the manuscript to align with eNeuro formatting requirements (e.g., figures removed from main manuscript file, figure legends placed after the references, etc). Line numbers below refer to those in the marked-up copy of the manuscript.

Major concerns 1. Due to the inherent limitations of cFos, a technique which identifies neural activity correlated with behavior, one cannot conclude that the cFos activated neurons drive squeaks or USVs. In order to claim that RAm contains three vocalization-related populations of neurons, the authors would ideally need to conduct perturbation experiments, for example, by combining TRAP2 experiments with loss of function perturbations (e.g., TeLC) demonstrating causal contributions.

Alternatively, in the absence of causal demonstration, the authors should adjust the language in the paper to reflect that these neurons are correlated/associated with the two vocal modes and avoid language that conflates correlation with causation. For example, in the second sentence of discussion (line number 475), the authors write that "USV production drives robust cFos expression". Similar language is used in multiple places in the results and discussion sections. It is recommended that these be adjusted to simply say that "cFos expression is correlated with USV production", in the absence of additional functional experiments.

We agree with the Reviewer, and we have corrected these instances (lines 229, 317-319, 357-358, 362-365, 420-423).

2. Do we know that all squeaks are emitted by female mice? This is a critical assumption underlying the experimental design. We understand that male mice produce the majority of USVs in a courtship context but could the authors cite relevant literature (e.g., in line 350) pointing out that courtship squeaks are produced predominately by females and not by males? Previous studies have also provided evidence that female mice produce the majority (and likely all) of the squeaks recorded during courtship interactions with males. In Wang et al., 2008, PLOS ONE, the authors 'silenced' either males or females by placing tape over their mouths. In pairs with a silenced female, male mice produced USVs but no squeaks.

Conversely, in pairs with a silenced male, females produced squeaks but no USVs. A number of previous studies (Wang et al., 2008; Grimsley et al., 2013: Finton et al., 2017; Klaus et al., 2025) have also reported that a large proportion of squeaks produced during male-female interactions overlap temporally with USVs, particularly during periods of male mounting. Given that males have been shown to produce the majority of USVs during courtship interactions with females and that mice cannot simultaneously produce USVs and squeaks, these studies support the idea that the majority of squeaks are produced by female mice during courtship interactions with males.

We now cite these studies in the Results section (lines 282-286), to provide additional rationale for our experimental design.

3. The rationale for using female-female interactions to elicit USVs in females rather than male-female interactions should be clarified. Should we assume that all USVs regardless of the context in which they are produced are associated with similar cFos expression in RAm? Past work employing a variety of methods (laryngeal nerve cuts, anesthetized mice, ultrasonic microphone arrays, mountable miniature microphones, and genetic ablation of neurons critical for USV production) has shown that males produce the majority of USVs during courtship interactions with females: • Anesthetized mice: Whitney et al., 1973 • Nerve cuts: White et al., 1998; Warburton et al., 1989 • ultrasonic microphone arrays: Heckman et al., 2017; Matsumoto et al., 2022; Neunuebel et al., 2015; Oliveira-Stahl et al., 2023; Sangiamo et al., 2020; Sterling et al., 2023) • mountable mics: Waidmann et al., 2025 • midbrain (PAG) neuronal ablation: Malone et al., 2025 In sum, female USV rates are low-to-negligible during courtship interactions, and even then, specialized methods would also be required to evaluate the total USVs produced by each subject female in this context.

Our goal was to maximize female USV production in order to elicit maximal and robust USV-associated Fos expression in RAm. We therefore opted to use female-female interactions to elicit USVs from female subjects. Past work has shown that single-housing females for a few days robustly elevates total USVs during female-female interactions (Zhao et al., 2021, Zhao et al., 2025). Although we did not employ methods to assign individual USVs to individual mice, the fact that single-housing also robustly increases social investigation and mounting in single-housed females suggests that at least many of the USVs are produced by the single-housed subject female. This paradigm thus ensures that our subject female produces a robust rate of USVs without the need for specialized USV localization methods. We have added language to the Results to better communicate this rationale (lines 247-255).

Robust rates of USV production elicit similar RAm Fos expression, at least for male courtship USVs and female-female USVs. This idea is supported by the similarity in spatial distribution of male courtship USV and female-female USV-associated RAm Fos expression (Fig. 1B-C). We also provide below for the Reviewers a direct comparison of total RAm Fos expression (summed across RAm sections 1-5) for our male USV and female USV Fos groups, which further emphasizes the similarity between these two groups (Reviewer Figure 1).

Reviewer Figure 1. Total USV time vs. total Fos-positive RAM neurons is shown for the male USV and female USV groups.

We think that the lack of such a pattern of Fos expression in the RAm of females that are given courtship interactions reflects their overall low USV rate in the courtship context. If it were possible to elicit a robust rate of USVs from females during courtship, we would hypothesize that the pattern of RAm Fos expression would look similar to the pattern associated with the production of male courtship USVs and female-female USVs. One caveat to that hypothesis is that because the acoustic features of female-female and male courtship USVs are highly similar (Hammerschmidt et al., 2012; Ivanenko et al., 2020), perhaps it is not surprising that USVs produced in these two contexts are associated with similar patterns of Fos expression in RAm. It remains undescribed whether any USVs produced by females in a courtship interaction differ acoustically from male courtship USVs or female-female USVs, but it is possible that USVs that differed acoustically from these might be associated with a different pattern of RAm Fos expression. In future work, it would be interesting to evaluate RAm Fos expression in a species that produce more acoustically diverse USVs (for example, one could evaluate RAm Fos expression in rats following the production of 22 kHz vs. 50 kHz USVs).

4. While manipulating interaction time to assign high-vocalizer vs. low-vocalizer groups is a reasonable strategy, it may also conflate with other non-vocal behaviors (such as locomotion, licking, grooming, etc) that may scale with interaction time. So, our concern is that neurons identified in Fig 1E may be involved in other non-vocal behaviors spuriously correlated with interaction time. Can the authors resolve this issue? One possibility would be to leverage the natural variability in vocalizations rates across animals. Could you show that the total number of cFos positive neurons scales with the number of vocalizations across animals that are allowed to interact for the same amount of time? For 2 of the 3 RAm Fos groups (male USV and female courtship squeak), we find that RAm Fos expression is positively and significantly correlated with total vocalization time, even when considering only mice that were given 30-minute-long trials. The figure below shows the relationship between total RAm Fos-positive neurons (considered for sections 1-5 for the male and female USV groups and for sections 4-5 for the female courtship squeak group) and total vocalization time. The left column shows the relationship for the full dataset from each group, and the right column shows the relationship separately for the short trial vs. long (30-minute) trial mice from each group.

Reviewer Figure 2. Total vocalization time vs. total Fos-positive RAm neurons is shown for the male USV, female USV, and female courtship squeak groups. Left column shows relationship for the full dataset for each group. Right column shows the relationship separately for short trial mice (orange datapoints) and long trial mice (blue datapoints). Statistically significant relationships are indicated in bold text.

In contrast to the male USV dataset, the female USV dataset lacks cases with intermediate total vocalization times, and so the relationship between total vocalization time and RAm Fos expression is relatively flat for the 30-minute female USV trials (middle row, blue datapoints in right column). Another caveat is that because both females in the pair can produce USVs, and because we did not employ methods to assign individual USVs to individual females, that total USVs produced by the subject female is not known precisely. Nonetheless, we think that the similarity in RAm Fos expression in the male and female USV groups and the overall strong relationships between RAm Fos expression and vocalization time in these groups support the idea that RAm Fos expression is associated with vocal production, rather than with a non-vocal behavior spuriously correlated with interaction time.

We now include these additional comparisons as Extended Data Figure 1-2, and we have adjusted the numbering of the other Extended Data figures accordingly.

Minor comments 1. While the authors mention the difference in functional context of squeaks vs. USVs in the introduction, a discussion on the mechanism of vocal production seems to be missing. USVs are produced by a whistle mechanism whereas squeaks are produced by vibration of the vocal cords. Could the authors clarify/speculate on how their result of differential recruitment of RAm neurons constrain the sound production mechanism? Given the difference in production mechanism between USVs and squeaks, one possibility is that largely non-overlapping sets of RAm neurons are recruited for the production of voiced vs. ultrasonic vocalizations. We have added this idea to the Discussion (lines 450- 454) 2. lines 54-55 seem to suggest that the different sets of muscles involved in vocalisation "overlap", which is clearly not the case: the differently involved RAm neurons may overlap, but the muscles do not! We did not mean to imply that the different muscles recruited during the production of different vocalization types are spatially overlapping (i.e., laryngeal, respiratory, and orofacial muscles are located in different parts of the body). Rather, we meant to say that certain muscles are likely to be recruited to some degree during multiple vocalization types. We have rephrased (line 31).

3. Please define "RAm voc neurons". Although this may seem intuitive it may not be for all readers.

We have clarified the wording as "these Fos-defined RAm neurons", rather than using the name "RAm-voc" neurons as in Park et al., 2024 (line 55-56).

4. For Fig. 3b, could the authors compare the observed proportion of squeak neurons that are also USV neurons (double positive) against the expected proportion if these two populations were simply independent. Under the independence model, the expected probability of double positive neurons is simply the product of the two individual probabilities: P(squeak)* P(USV) = P (squeak ∩ USV). This would provide quantitative evidence of whether the proportion of double positive neurons is simply expected by chance, consistent with their hypothesis of USVs and Squeak recruiting independent neuronal populations in the RAm.

We agree with reviewer that the independence model could in principle be used to test whether the proportion of double-labeled neurons (either USV-squeak or squeak-USV) differs from the overlap expected by chance. One uncertain but important piece of information for this calculation is the total number of RAm neurons. Veerakumar et al. (2023) estimated that there are 160 NTS+ RAm neurons, intermingled with 1100 NTS- negative RAm neurons. We could then assume that there are ~1300 total RAm neurons. Because we considered approximately every other coronal section in our analysis, we would then estimate that there are a total of ~650 total neurons across sections 1-5 of RAm. If these neurons are distributed evenly across sections 1-5, we would estimate ~130 RAm neurons in each coronal section. However, these numbers don't align well with our mean counts of Fos-positive neurons. For example, in our male high USV group, the mean number of Fos-positive neurons across sections 1-5 is: 102.8, 98.5, 101.8, 104.3, and 91.5. At face value, that would mean that approximately 75% of total RAm neurons were Fos- positive in association with male USV production. The relative density of Fos-positive neurons in our image doesn't seem consistent with that value, which would lead us to believe that either the total rostral-to-caudal extent of RAm and/or the size of ROI we drew to demarcate RAm in each section is larger than that considered by Veerakumar et al.

Unfortunately, we did not collect our confocal images in a way that would allow us to perform accurate total cell counts from each RAm section using the Neurotrace labeling (we collected single confocal images rather than z-stacks from relatively thick coronal sections), in order to independently arrive at an estimate of total RAm cell count across the rostral-to-caudal extent of RAm that we considered and using the ROIs as we drew them.

Even without knowing the total number of RAm neurons within the rostral-to-caudal extent that we considered, the independence model would predict higher overlap in sections 4-5 than in sections 1-3, because P(squeak) is highest in the caudal-most RAm sections. No such trend is present in our data, however, which provides perhaps some evidence that our overlap data diverges from the pattern one would expect for two entirely independent populations. Given the limitations of our dataset to perform the type of calculation, though, we prefer to leave this discussion out of the manuscript.

5. In line 493 'that' should be 'than' since a comparison is involved.

Thank you for catching this typo.
